# The Role of Temporal Modulation in Sensorimotor Interaction

**DOI:** 10.3389/fpsyg.2019.02608

**Published:** 2019-12-06

**Authors:** Louis Goldstein

**Affiliations:** Department of Linguistics, University of Southern California, Los Angeles, CA, United States

**Keywords:** speech production, temporal modulation, articulation, acoustics, syllable structure, sensorimotor interaction

## Abstract

How do we align the distinct neural patterns associated with the articulation and the acoustics of the same utterance in order to guide behaviors that demand sensorimotor interaction, such as vocal learning and the use of feedback during speech production? One hypothesis is that while the representations are distinct, their patterns of change over time (temporal modulation) are systematically related. This hypothesis is pursued in the exploratory study described here, using paired articulatory and acoustic data from the X-ray microbeam corpus. The results show that modulation in both articulatory movement and in the changing acoustics has the form of a pulse-like structure related to syllable structure. The pulses are aligned with each other in time, and the modulation functions are robustly correlated. These results encourage further investigation and testing of the hypothesis.

## Introduction

Work over the last 20 years has revealed abundant evidence for real-time sensorimotor interaction in both speech production and speech perception. In speech production (the topic of this volume), the role of auditory feedback in guiding speech production has been demonstrated in experiments showing that talkers may produce compensatory articulatory changes in response to altered auditory feedback (Houde and Jordan, 1998). In addition, talkers can align their articulatory patterning in real-time to that of a partner, in the so-called “synchronous speech” task (Cummins, 2002). While less obviously real-time, talkers have also been showed to alter the temporal profile of their articulation to match that of a partner in experiments showing phonetic convergence (e.g., Lee et al., 2018). More generally, of course, vocal learning requires the ability to use auditory information to guide changes in articulatory behavior.

The existence of such sensorimotor interactions would appear to require that speakers have some common representation of speech articulation and acoustics that affords the kind of alignment that these experiment results exhibit. At first blush, it is tempting to think that evidence for this common representation might be found in the neural activation patterns in the motor cortex like those that have been found during listening to speech (Wilson et al., 2004). Indeed, the dual-stream model (Hickok and Poeppel, 2007) hypothesizes that such neural activation subserves sensorimotor control of speech production. However, recent experiments using electrocorticography have shown that the representation of speech segments in the motor areas during listening is quite distinct from its representation in the same areas during speech production. Cheung et al. (2016) compared the activation patterns while patients produced CV syllables and while they listened to recordings of themselves producing those syllables. The activation patterns of speech segments in the anterior ventral sensorimotor cortex (vSMC or “motor cortex”) during listening was found to be organized by their acoustic properties, clustering segments by manner classes, as is also found in the auditory areas such as the superior temporal gyrus and others (Mesgarani et al., 2014). However, activation patterns during speaking were found to be organized by vocal constricting organ (labial, coronal, dorsal), consistent with other recent work that has shown that electrode activity can be predicted as a function of coordinated articulatory movement creating constriction gestures of those three types (Chartier et al., 2018). Thus, the patterns of neural activation associated with acoustics and articulation of the same utterance are distinct, even in the motor areas. So, what binds them together to afford their interaction or integration?

Like most work contrasting articulatory versus acoustic representations in speech production and perception (and in phonology), the research described in the previous paragraph focuses on the paradigmatic aspects of the neural representations, e.g., how the neural representations of distinct speech segments differ in the same context. However, this focus ignores the temporal aspects of continuous acoustic and articulatory signals, which must be lawfully related as the articulatory movements actually cause the acoustic signals. Temporal aspects of the corresponding cortical representations have been the focus of recent work by Assaneo and Poeppel (2018) who found that cortical oscillations in auditory and speech-motor areas are synchronized with one another during listening, specifically to syllable repetition rates around 4.5 Hz, and have proposed this synchronization as a possible solution to the binding problem. Their model of the synchronization involves entrainment of theta-band (4–8 Hz) oscillations in the auditory cortex to the speech envelope as has been shown in other recent work (Doelling et al., 2014), as well as the coupling of neural oscillators in the auditory and speech-motor areas. In this listening situation, rhythmic regularities of the acoustic speech envelope in the theta band plays a key role in the entrainment model, and they have also been shown to contribute to the intelligibility of the speech (Ghitza and Greenberg, 2009) and to listener sensitivity in detecting gaps in artificial stimuli with speech-like rhythmic properties (Henry et al., 2014). However, turning to speech production, it is unknown whether there are periodic components in ongoing articulatory-motor activity that could play a role like that of the speech envelope in entraining cortical oscillations and contribute to synchronization of auditory and speech-motor areas. This may be due to the difficulties in obtaining “clean” brain responses from talking participants (both in the MRI scanner and during EEG acquisition) and provides a motivation for probing the temporal modulation of speech articulation and its relation to acoustic modulation.

The temporal dimension of the articulatory and acoustic structure of speech is the focus of the work to be described here. This work hypothesizes that there should be a systematic relation between the temporal modulation of articulation (*how much* is it changing at any given moment) and the corresponding temporal modulation of the acoustic signal, specifically ignoring in what way the signals are changing.

The cognitive significance of patterns of modulation or change over time has been addressed in a variety of domains. For example, viewers can perceive humans engaging in a variety of actions when watching dynamic point-light displays (e.g., Rosenblum et al., 1996), but there may be nothing in the static displays of the dots to suggest different human body parts or their similarity structure. Sinewave approximations to human speech (Remez et al., 1981), which were loosely modeled on point-light displays, preserve information about how frequency information in the signal changes over time, but static moments of the signal may not be so readily perceived as speech.

Measures of change over time have been incorporated into automatic speech recognition systems through use of the modulation spectrum (e.g., Kingsbury et al., 1998) or by using the derivatives of acoustic measures, such as Mel-frequency cepstral coefficients (mfccs), as additional feature vectors (Furui, 1986). Derivatives have also been incorporated into some approaches to acoustic-to-articulatory inversion (Ghosh et al., 2009; Mitra et al., 2012). However, the structure of the modulation patterns in articulation and acoustics and their alignment have not been systematically or quantitatively investigated, nor has the potential relation of those modulation patterns to phonological structure. A first step at such an investigation is the goal of this paper.

The investigation takes as input temporal modulation functions of articulation and acoustics derived for utterances drawn from the X-ray Microbeam Speech Production Database (Westbury et al., 1994). Of necessity, the investigation is largely exploratory, as such modulation signals have not been explicitly investigated previously. Nonetheless, the main underlying hypothesis is that the modulation functions should be systematically correlated in some fashion. In addition, consideration of what is generally known about the structure of speech leads to some expectations, or predictions (in a loose sense), about the nature of these functions and their correlation.

We know that the speech signal does not change in a continuous way but rather is temporally structured. There are intervals of time, such as during a long, stressed vowel, during which the articulation and acoustics are not changing very rapidly, and there are other intervals, such as at the time of release of an onset consonant into a vowel or at the formation of a coda consonant, when change is rapid. Sharp acoustic change is seen in discontinuities in a spectrogram that are used as acoustic segmentation criteria for durational measurement. At the level of articulatory kinematics, several gestures are proceeding in close temporal sequence at release of an onset consonant, for example: release of the consonant constriction gesture, production of the vowel gesture, adduction of the vocal folds if the consonant is voiceless, lowering of the velum if the consonant is nasal (see Tilsen and Goldstein, 2012). This leads to two predictions: (1) Modulation functions of both articulation and acoustics should exhibit a pulse-like structure, alternating between periods of rapid change (change “pulses”) and periods of little change. (2) The period of repetition of the pulses should be related to the syllable repetition rate, with one to three pulses per syllable depending on its complexity: one pulse in a simple CV syllable, somewhere between the onset consonant’s release and the vowel, and additional pulses if the syllable has one or more coda consonants. Considering next the relation between the articulatory pulses and the acoustic ones—further predictions can be made: (3) Since articulatory change generally gives rise to acoustic change, there should be robust correlations between the articulatory and acoustic modulation functions, which have not been systematically evaluated in the past. One possible source of the correlations is that over the course of running speech, prosodic structure influences the velocity of articulator movements, such that velocities are slower near boundaries (Edwards et al., 1991). This slowing should be observable in the magnitudes of the modulation functions, both articulatory and acoustic. If this were the *only* source of correlation, it would suggest that spans of speech long enough to include prosodic phrase boundaries would be required in order for the system to solve the binding problem, which might not be realistic. It is important, therefore, to investigate the correlations in temporal windows of different length. (4) Finally, the temporal locations of articulatory and acoustic modulation maxima (pulses) should be systematically aligned. To the extent that speech has a rhythmic structure (Tilsen and Arvaniti, 2013; Lancia et al., 2019), the pulses observed in both modulation functions should have a repetitive structure, and that repetitive structure should be shared across the two functions.

## Materials and Methods

### Data

The study described here is a secondary analysis of publicly available, already published data from the X-ray Microbeam Speech Production Database (Westbury et al., 1994). For the analysis here, one sentence from the database was selected from one of the read paragraph tasks that the participants performed (the ‘Hunter’ paragraph): *Once he thought he saw a bird, but it was just a large leaf that had failed to drop to the ground during the winter.* Of the participants who recorded this sentence, 23 were selected (15 female and 8 male) who read the sentence with no audible hesitations and with only a single pause (after “bird”). The speakers were all students at the University of Wisconsin in the early 1990s. Their Dialect Base (described in Westbury et al., 1994, as “place of residence during linguistically formative years”) included 13 from Wisconsin, 3 from Illinois, 2 from Minnesota, and one each from Indiana, Colorado, California, Massachusetts, and New Jersey. The data analyzed include markers attached midsagittally to the upper lip (UL), lower lip (LL), lower incisor (LI), four tongue markers (tip to dorsum: T1, T2, T3, T4), and simultaneous audio.

Pause durations following the word “bird” were measured manually from a wide-band spectrogram, from the release of the final/d/in “bird” to the release of the initial/b/in “but.” The average syllable duration for each speaker’s production was estimated by taking the duration of the entire sentence for a given speaker, subtracting the pause duration (following “bird”), and dividing the result by the number of syllables (*n* = 27).

### Articulatory Modulation Functions

Articulatory change, or modulation, was defined for a given frame as the sum of the squared velocities of the 14-dimensions defined by the 7 markers × 2 dimensions (*x*,*y*), as in (1):

(1) $$M\,B\,E\,A\,M\,\left(k\right)=\sum_{i=1}^{7}\sum_{j=1}^{2}{\left(m\,\left(i,j,k+1\right)-m\,\left(i,j,k\right)\right)}^{2}$$

where *m(i,j,k)* are the marker positions for marker (*i*) 1-7 (UL, LL, T1, T2, T3, T4, LI), dimension (*j*) 1-2 (*x*,*y*), at frame *k*. Ignoring the mass of the articulators (i.e., treating all masses = 1), MBEAM also is twice the kinetic energy of the set of articulators (KE = 0.5 mv^2^).

**FIGURE 1:**
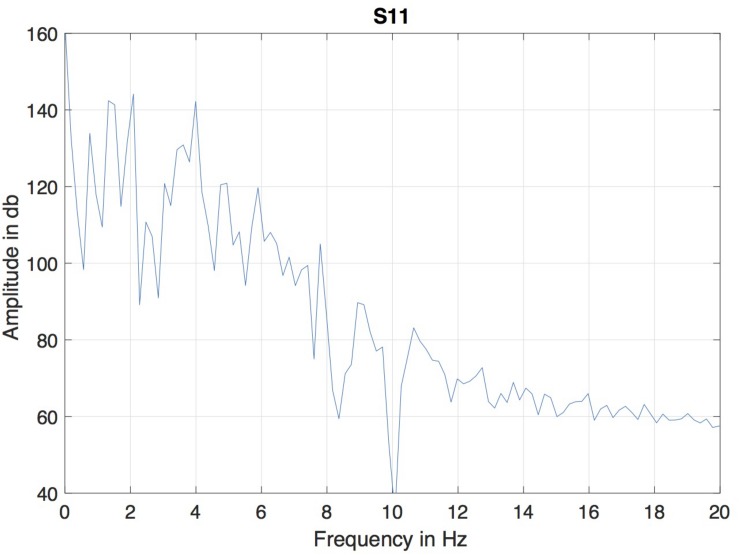
Magnitude spectrum of the vertical position of T1 (marker nearest the tongue tip) for the test sentence produced by of the speaker (S11).

A version of microbeam corpus in Matlab format was employed. In this format, the data of all markers was interpolated to a fixed sampling rate of 145.6 Hz, so the duration of each frame was 6.866 ms. Because of the differencing involved in computing the MBEAM function, it is effectively high-pass filtered and can be noisy. The resulting MBEAM functions were therefore smoothed. To determine the appropriate frequency cutoff for the smoothing filter, the frequency content in the microbeam marker signals themselves was considered. Since the tongue tip marker was acquired at the shortest original (nominal) sampling period during the data acquisition (before the acquired data were interpolated by a smoothing spline to make samples all equal in duration, Westbury et al., 1994), the magnitude spectrum of the vertical movement of the marker closest to the tip of the tongue (T1) for the test sentence produced by each of the speakers was examined. The results of a typical speaker are shown in Figure 1. For all speakers, the amplitude of the spectrum at 10 Hz is down by 60 dB from its peak value, and changes little at higher frequencies. A cutoff frequency of 12 Hz was chosen, and the MBEAM functions were filtered in Matlab (Mathworks, Inc.) using a zero-phase, low-pass, nine-point Butterworth filter with a 12 Hz cutoff. In order to test if the resulting filtering overly determines the correlation results, another version of the MBEAM functions was created using a 25 Hz cutoff filter, and analyses were replicated using these functions.

The temporal structure of each MBEAM function was characterized by finding the times of the successive maxima of the function (using the zero-crossings of its derivative). These maxima will be referred to as the *modulation pulses.* The mean inter-pulse interval and its standard deviation were calculated. An alternative would be to define pulses as the maxima of the derivative of the modulation function, i.e., acceleration maxima where the velocity is changing most rapidly and which can be thought of points of maximum force, but this was not explored in this work. In order to test the predictions about the relation between pulses and syllable structure, the segment and word transcriptions of the 23 sentences were aligned to the audio signals using the Penn Forced Aligner (Yuan and Liberman, 2008). The segmentations were checked by hand and corrected where necessary. Almost all errors involved the low intensity fricative in “failed,” which was often mistakenly characterized as a short pause. Since all but two of the words (“during” and “winter”) were monosyllabic, the word-level segmentation also served as a syllable segmentation. “During” was divided into syllables between the [r] and the [I], and “winter” between the [n] and the [t]. For each syllable of the transcription, the number of pulses falling in the temporal window of that syllable was automatically tallied. For each speaker, the mean number of pulses falling on open syllables (no coda consonant), syllables with single coda consonants, and syllables with more than one coda consonant were calculated.

### Acoustic Modulation Functions

The signal representation chosen as the basis of the acoustic modulation functions is a set of mel-frequency cepstral coefficients (mfcc). In addition to fact that this representation has been widely used in speech technology applications (Rabiner et al., 1993), it encodes the resonance structure of the vocal tract, but not voiced source fundamental frequency, which of course is also not captured by microbeam markers on the surfaces of the vocal tract. Mfccs have been used in work that has successfully estimated articulator point marker time functions from acoustics using deep neural nets (Chartier et al., 2018) and other techniques (Mitra et al., 2011; Afshan and Ghosh, 2015). Mfcc parameters were calculated for the audio signals paired with the microbeam data using Matlab code developed by Kamil Wojcicki and available on the Mathworks File Exchange^1^. The window size for the analysis was 25 ms, and time between frames was chosen to be equal to the frame rate of the MBEAM functions, i.e., 6.866 ms. The audio signal was preemphasized using a high-pass filter (coefficients [1, −0.97]), analyzed using 20 filterbank channels over the frequency range 0–3,700 Hz, as changes in this frequency range can be expected to be well-determined by changes in the anterior articulator positions that do not produce the narrow constrictions associated for example with fricatives. The spatial representation of such narrow constrictions is expected to be poorly related to fricative acoustics due to the potential mechanical interaction of the marker with the palate. 13 mfcc parameters were extracted, similar to the dimensionality of the microbeam data.

As the bandwidth of the unsmoothed mfcc parameters may be considerably higher than that of the microbeam markers, each coefficient was filtered using the same (12 Hz) smoothing filter used preceding calculation of the MBEAM modulation function. The (MFCC) modulation function was calculated as in (2) in a similar manner as the MBEAM modulation function:

(2) $$M\,F\,C\,C\,\left(k\right)=\sum_{i=1}^{13}{\left(f\,\left(i,k+1\right)-f\,\left(i,k\right)\right)}^{2}$$

where *f(i,k)* represents the *i*th mfcc at frame *k*. Due to the resulting high-pass filtering, the resulting MFCC function was also smoothed using a zero-phase, low-pass, nine-point Butterworth filter with a 12 Hz cutoff. As with the MBEAM functions, another version was created using a 25 Hz filter. The mean inter-pulse interval and its standard deviation were calculated in the same way as for the MBEAM function, and the mean number of pulses per syllable type for each speaker was calculated in the same way as for the MBEAM pulses.

### Correlation Methods

In order to test the predictions that (a) there are robust correlations between articulatory and acoustic modulation functions and that (b) there is a repetitive temporal structure shared between articulatory and acoustic modulation functions, Correlation Map Analysis (CMA) was employed (Barbosa et al., 2012; Gordon Danner et al., 2018). CMA calculates a correlation time function between two signals using a sliding window centered on each sample of the signals. The window is actually a kernel: every sample in the signals contributes to the correlation, but the contribution of samples to the correlation decreases as a function of lag from the center of the window, as determined by a weighting function. (3) Shows the expression for calculating a covariance function between two signals *x* and *y*, at every sample (*k*).

(3) $$S_{x\,y}\,\left(k\right)=\sum_{l=-\infty }^{\infty }c\,e^{-\eta \,\left|l\right|}\,x\,\left(k-l\right)\,y\,\left(k-l\right)$$

*l* is the sample lag from the center of the window, and η (eta) is the parameter that determines the sharpness of the window. Greater values of η result narrower time windows. *c* is a constant chosen so that the sum of the weights over all samples is 1. Correlation (ρ) at each sample is then calculated as in (4).

(4) $$\rho \,\left(k\right)=\frac{S_{x\,y}\,\left(k\right)}{\sqrt{S_{x\,x}\,\left(k\right)\,S_{y\,y}\,\left(k\right)}}$$

The choice of η determines an effective frequency cutoff of the resulting correlation time function, for which Barbosa et al. (2012) provide an approximation function. Three values of η were chosen: a narrow window (η = 0.8) that produces a frequency cutoff of 12.4 Hz (roughly equal to cutoff frequency of the modulation functions themselves), a wide window (η = 0.08) that has a much lower frequency cutoff (1.24 Hz), and an intermediate value (η = 0.2) with a frequency cutoff of 3.1 Hz. For each value of η, the median of the correlation values across all the samples in the correlation function for a given speaker was calculated.

In order to provide a baseline with respect to which the observed correlation values can be evaluated, surrogate signal pairs where created, in which there is no systematic causal relation between the values of two signals. To create a surrogate pair, the *k* samples of each MFCC modulation function were divided into two halves (first and second *k/2* samples), the order of the two halves was then reversed, and the resulting signal was paired with the unchanged MBEAM function. As a result, the first half of the MBEAM function was paired with the second half of the MFCC function, and second half of the MBEAM function was paired with the half of the MFCC function (Note that the same result would have been achieved by reversing halves of the MBEAM function). Any remaining correlation between the surrogate signals reflects general properties of signals of this type (as calculated with this method), not a causal relation between the two signals. The surrogate signal pairs were analyzed using the same conditions of filtering and η as used with the original signals. For each value of η, the median of the correlation values across all the samples in the correlation function for the original signal pairs was compared with the median values obtained with the surrogate pairs.

Correlation map analysis also calculates the correlation functions between signals as they are shifted in time with respect to each other. Critically, this allows us to evaluate the hypothesis that there is a repetitive temporal structure to the modulation pulses shared between the articulatory and acoustic functions. One way of characterizing the repetitive (or periodic) structure of a single signal is to examine the autocorrelation function of the signal, which represents the signal correlated with itself at different lags. To the extent that the signal has a periodic structure, there will be a clear peak in the autocorrelation function at a non-zero lag corresponding to the fundamental period of repetition. The autocorrelation functions of the MBEAM and MFCC functions were calculated individually using CMA to compare the signals with themselves at different lags, and the period of the repetition associated with each was determined by finding the lag associated with the maximum median correlation of the correlation function (other than zero-lag, which in the case of correlating a signal with itself always yields a correlation equal to 1). To evaluate the shared repetitive structure of the MBEAM and MFCC functions, the median correlation of MBEAM and MFCC at lags from −200 to +200 ms were compared to find the lags at which the correlation is maximal. The zero-lag is predicted to be maximal, because at this lag, the acoustic change at a given frame is aligned in time with the articulatory change that caused it. The changing shape of the vocal tract causes an immediate change in its acoustic source and filter properties. If there is any delay at all, it is much shorter than the 6.86 ms frame duration. If the form of the function relating lag to correlation has the form of an autocorrelation function, it will also be possible to find robust secondary maxima in the function. At the lag corresponding to a secondary maximum, the articulatory change is not aligned in time with the acoustic change that it caused, but the repetitive structure of the signals is such that articulatory modulation pulses (maxima) are still aligned with acoustic modulation pulses, and frames with little articulatory modulation are aligned with frames of little acoustic modulation. This is then the period of shared repetitive structure for the pair of signals. These values will be compared against the single-signal autocorrelation functions.

## Results

### Characterization of Modulation Functions

**FIGURE 2:**
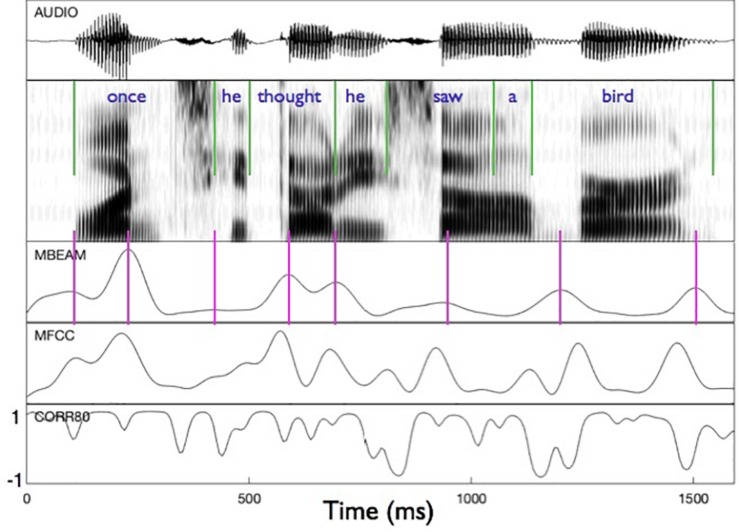
Sample of modulation functions and their correlation function for the first clause of the test sentence, for one of the speakers. Panels represent (from **top** to **bottom**): audio waveform, sound spectrogram, MBEAM modulation function filtered at 12 Hz, MFCC function filtered at 12 Hz, and the correlation function from Correlation Map Analysis for the narrow window condition, η = 0.8. Green vertical lines represent acoustic segmentation into syllables. Purple vertical lines mark the peaks of the MBEAM function.

Figure 2 shows an example of the MBEAM and MFCC modulation functions (obtained with the 12 Hz filtering) along with the correlation function resulting from the CMA analysis (for η = 0.8) in the bottom panel. The first clause of the test sentence is shown (both waveform and spectrogram) for one of the 23 speakers. The pulse structure of the MBEAM function is obvious from the figure. As expected, the pulse peaks (times of maximum articulatory change; shown with vertical magenta lines) align reasonably well with points of rapid or discrete change in the spectrogram. Two peaks are found during the syllable corresponding to “once,” one peak for “he,” one for “thought,” etc. The MFCC modulation function exhibits a similar structure, although it has more peaks than the MBEAM function. This is reasonable, as there is more information in the MFCCs than in the MBEAM and it is more fine-grained temporally: source changes and nasalization are not represented in the MBEAM data, and it is derived from measurements of the anterior tract only. But for every MBEAM peak there is an MFCC peak close in time to it. Typically, the MBEAM peak lags the MFCC peak (except in “bird”). Presumably this is due to the fact that the MFCC frames are based on 25 ms windows and so “look ahead” of the corresponding MBEAM frame. Overall, the correlations shown in the bottom panel are quite high, with the clear majority of points showing positive correlations.

**FIGURE 3:**
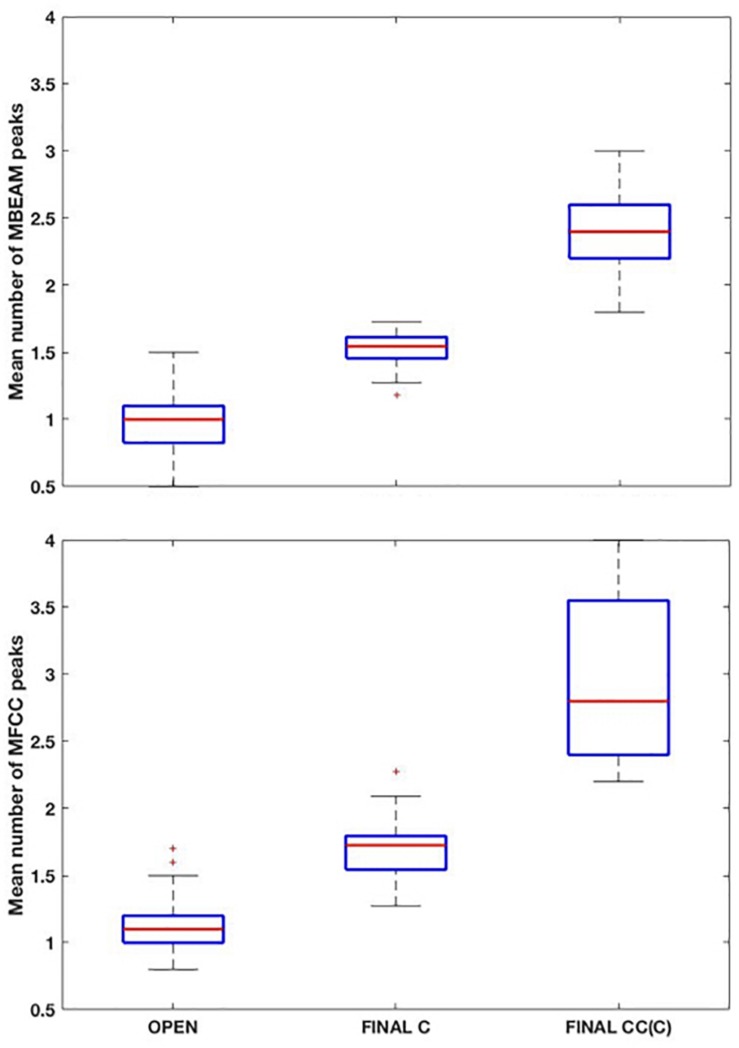
**(Top)** Box plots of the mean number MBEAM peaks (pulses) per syllable as a function of syllable type: “open: (no coda consonant), “final C” (single coda consonant), “final CC(C)” (two or more coda consonants). Each speaker contributes a single value to each box plot, which is the mean number of peaks found in syllables of that type for the speaker. **(Bottom)** Same plots as **top panel**, but for MFCC peaks.

Box plots showing the mean number of MBEAM pulses occurring during open syllables, syllables closed by a single consonant, and syllables closed by more than one consonant are shown in the top panel of Figure 3 (again for the 12 Hz filtering condition). Each speaker contributes one mean per box plot. As predicted, the mean of the open syllables is close to 1 (0.97), while the mean of syllables closed by a single consonant is 1.51, possibly suggesting that half the syllables have two pulses while the other half have only one. The difference between these two syllable types is highly significant (sign test *p* < 0.001), as 22 of the 23 speakers have more pulses in the case of the coda condition. (Here and in all the sign tests performed, *p*-values obtained that are less than 0.001 are reported as *p* < 0.001). Finally, the mean of syllables with more than one coda consonant is almost twice the mean with a single coda (2.4). The differences between one and two coda consonants is likewise highly significant (*p* < 0.001), as all speakers have more pulses with multiple codas. The pattern of results for MFCC pulses are very similar but with a few more pulses overall, as shown in the bottom panel of Figure 3. The means for the three conditions are 1.15, 1.69, and 2.91, and the differences are highly significant in a sign test.

**FIGURE 4:**
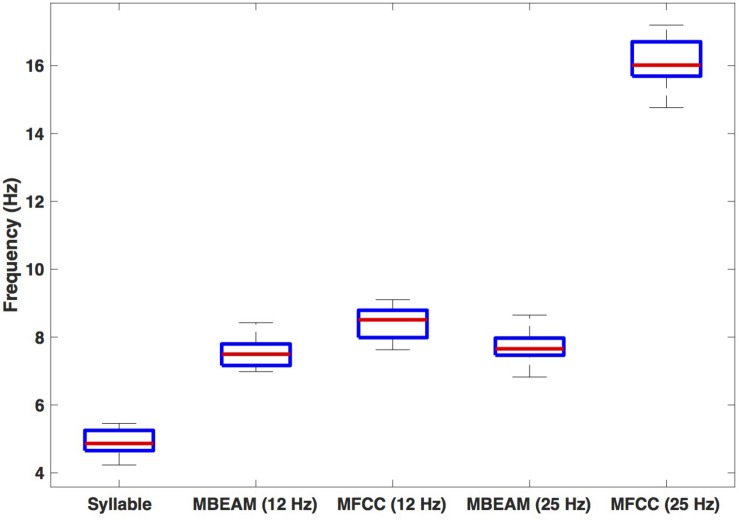
Box plots of estimated frequencies of: syllables, MBEAM pulses, and MFCC pulses, for 12 and 25 Hz filter conditions. Each speaker contributes a single value to each boxplot.

Figure 4 shows box plots for the mean frequency of the 23 speakers’ inter-pulse intervals for the MBEAM and MFCC functions (calculated from the mean inter-peak durations) for both 12 and 25 Hz smoothing conditions. Also shown are the syllable frequencies, calculated from the mean syllable durations. Examining the 12 Hz results, the MFCC frequencies are higher than the MBEAM frequencies (not surprisingly, since there are more MFCC pulses than MBEAM pulses), and the difference is highly significant (*p* < 0.001) in a sign test across the 23 speakers (all but two show higher MFCC frequencies). It is also clear that both of those frequencies are higher than syllable frequency. The median syllable frequency is 4.9 Hz and the median MBEAM frequency is 7.5 Hz. Their ratio is 1.5, which is consistent with the results in Figure 3, showing about one pulse per open syllable, but more pulses for syllables with coda consonants. Considering the results for the 25 Hz smoothing, the MBEAM frequencies are basically unchanged from the 12 Hz condition (median frequency for the 25 Hz condition is 7.7 Hz); the difference is not significant by sign test. Thus, the 7.5 Hz inter-peak frequency value for MBEAM modulation function data appears to characterize the temporal modulation in these (relatively slowing changing) articulatory signals quite well. The MFCC inter-peak modulation frequency is obviously much higher in the 25 Hz condition than in the 12 Hz condition (16 vs. 8.5 Hz). The 12 Hz filtering has removed higher modulation frequencies that are contained in the faster-changing acoustic signals and smoothed it to make it more comparable to the MBEAM function.

### Correlation Analysis

#### Surrogate Analysis and Window Width

**FIGURE 5:**
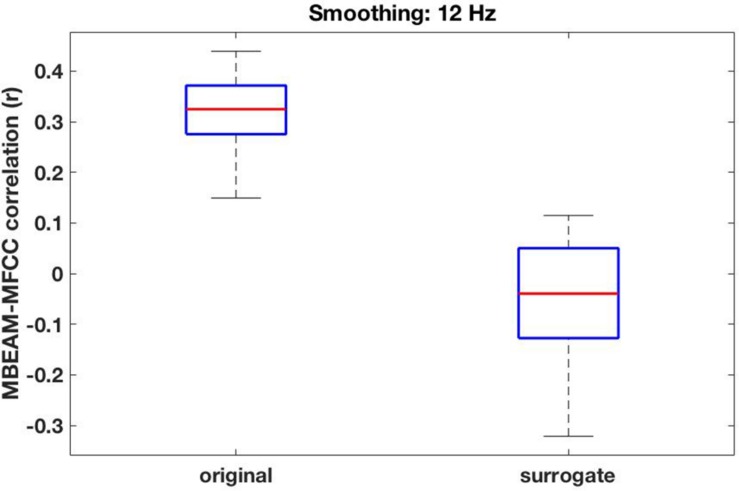
Box plots of the overall global correlation values between the MBEAM and MFCC modulation functions—filtered at 12 Hz—for the 23 speakers, original data on the **left**, surrogate data on the **right**.

**FIGURE 6:**
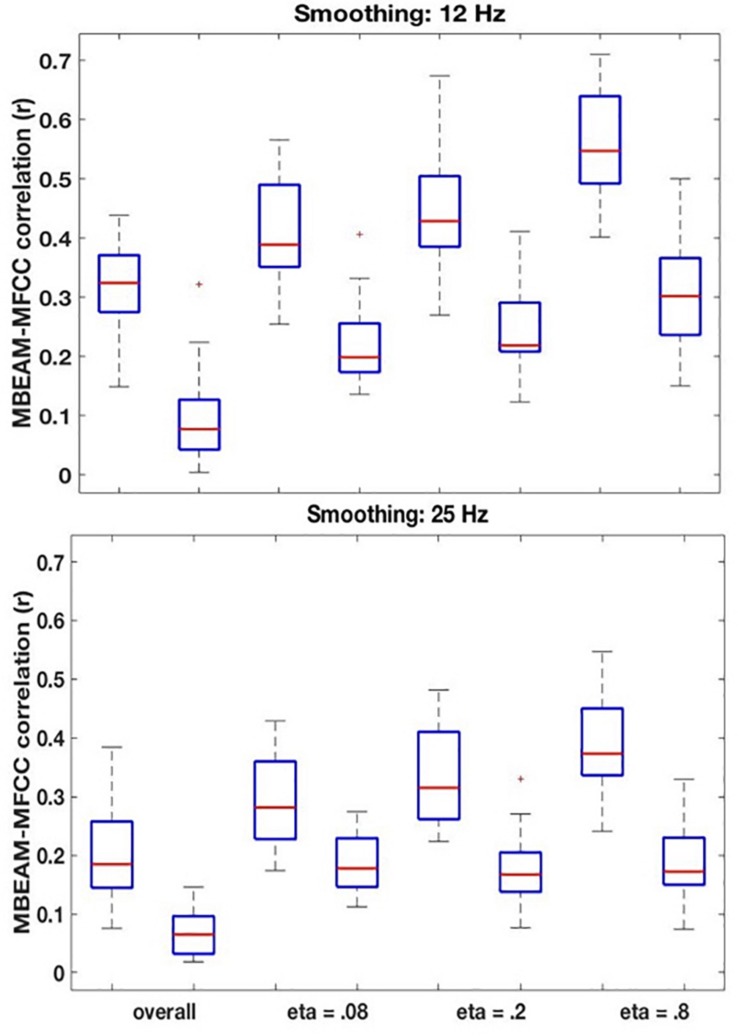
**(Top)** Box plots comparing correlations between the original MBEAM and MFCC modulation functions filtered at 12 Hz and the correlations of the corresponding surrogate data functions, for four different correlation types: the overall correlation and the median values of the CMA correlation function for three different values of η. For each of the correlation types, the original data is plotted on the left and the surrogate data on the right. In all cases, the absolute values of the correlations are plotted. **(Bottom)** Same plots as **top**, for modulation functions filtered at 25 Hz.

For the 12 Hz filtering condition, the global (‘overall’) correlation of the MBEAM and MFCC functions is positive and significant for every speaker (*p* < 0.001). The box plot of the 23 correlation values is shown in the left plot in Figure 5. For the surrogate data plotted on the right, only 12 speakers show significant correlations (significance varying from *p* < 0.05 to *p* < 0.001) and of those 8 are negative and 4 are positive. Because so many of the surrogate pairs are negatively correlated, comparison of the original and surrogate data is most conservatively done with the magnitudes of original and surrogate correlations, i.e., taking the absolute values of the surrogate correlations. Box plots of the resulting values are shown in the leftmost pair of columns of Figure 6 (top panel); original on the left, surrogate on the right. A sign test confirms that the magnitude of the correlations is higher for the original than for the surrogate data (*p* < 0.001). All speakers but one (S34) have higher magnitude correlations in the original data. S34’s surrogate correlation is in fact negative.

The remaining boxplots in Figure 6 (top panel) plot the results of the CMA analysis for the three values of η. For each value, the results of the original data are plotted on the left, and the surrogate data on the right. For each value of η, the median value of the correlation function from the CMA analysis for each subject was calculated for each signal lag, and the maximum positive correlation value and the maximum negative correlation of that median across the lags was determined. The lag with the higher magnitude was taken to represent the correlation for that speaker, and is plotted in the box plots. For every value of η, a sign test confirms that the magnitude of the original data correlation is higher than that for the surrogate data (*p* < 0.001). There are two other ways in which the original data correlations exhibit a strikingly different pattern of results than the surrogate data. First, for the original data, for every value of η and for every speaker (except for speaker S30 for η = 0.8), the maximum positive correlation was higher in magnitude than the maximum negative correlation. However, for the surrogate data, a sign test revealed that there was no tendency for the highest magnitude correlation to be either positive or negative. Second, the lags that show the maximum positive correlations for the original data are tightly clustered around 6.866 ms (or a one frame delay of the MBEAM signal)^2^, with very small standard deviations, as shown in Table 1. The lags at which the maximum correlations (positive or negative) occur for the surrogate data are much more variable; the standard deviations of these lags are an order of magnitude higher than for the original data. Thus, the original data show robust, positive correlations between MBEAM and MFCC functions when the signals are temporally aligned with close to zero lag. The correlations exhibited by the surrogate data are weaker and are variable both in sign and in the lag at which the highest magnitudes are found.

**TABLE 1:** Medians and standard deviations (across speakers) of the lag (in ms) at which the highest positive and negative correlations are found between MBEAM and MFCC functions.

		**Positive r**		**Negative r**	
			
		**Median**	***SD***	**Median**	***SD***
η = 0.08	Original	6.9	7.3	75.5	126.6
	Surrogate	20.6	98.3	–34.3	114.1
η = 0.2	Original	13.7	5.8	48.1	94.9
	Surrogate	20.6	101.9	–13.7	105.8
η = 0.8	Original	6.9	7.3	–48.1	68.0
	Surrogate	27.5	92.3	–27.5	99.6

*Results shown separately for original and surrogate data as a function of eta. 12 Hz smoothing condition.*

As can also be seen in Figure 6, the results show that the correlation is higher in narrower time windows than in wider ones. The correlation values for the original data show a regular progression as a function of window size—η = 0.8 > η = 0.2 > η = 0.08 > overall. The difference between each of the adjacent steps in the progression was tested in three sign tests, and each is significant (at least *p* < 0.005). However, the same trend is found with the surrogate data, and the difference between the overall correlation and η = 0.08 is significant in a sign test, as is the difference between η = 0.8 and η = 0.20. Thus, the differences between narrow and wide windows may be due to some aspect of the method, rather than being informative of the locus of the correlation between the functions. However, the results clearly demonstrate that a wide (i.e., temporally long) window is not necessary to obtain meaningful correlations.

The results for the 25 Hz filtering condition are shown in the bottom panel of Figure 6. The correlations are lower than those in the top panel, as expected given the increased number of MFCC pulses in this condition. Nonetheless, the overall pattern of results is the same as for the 12 Hz filtering condition. A sign test confirms that the magnitude of the correlations is higher for the original than the surrogate data for the overall correlation and for all values of η (*p* < 0.001, except for η = 0.08, *p* = 0.011). As was the case for the 12 Hz condition, all speakers showed positive overall correlations for the original data, but there was no cross-speaker tendency for the sign of the correlation in the surrogate data. In the CMA analyses, for the original data, for every value of η the maximum positive correlation was higher in magnitude than the maximum negative correlation for a significant number of subjects (*p* < 0.005). However, for the surrogate data, a sign test revealed no tendency for the highest magnitude correlation to be either positive or negative. Likewise, as is shown in Table 2, the lags that exhibit the maximum positive correlations for the original data are clustered around 6.866 ms, with relatively small standard deviations; while lags at which the maximum correlations (positive or negative) occur for the surrogate data are much more variable.

**TABLE 2:** Medians and standard deviations (across speakers) of the lag (in ms) at which the highest positive and negative correlations are found between MBEAM and MFCC functions.

		**Positive r**		**Negative r**	
			
		**Median**	***SD***	**Median**	***SD***
η = 0.08	Original	6.9	49.6	–48.1	82.7
	Surrogate	–20.6	121.0	–89.3	111.0
η = 0.2	Original	6.9	24.7	–48.1	91.6
	Surrogate	6.9	92.6	–61.8	111.0
η = 0.8	Original	6.9	28.3	–48.1	71.4
	Surrogate	–6.9	111.5	–55.0	111.3

*Results shown separately for original and surrogate data as a function of eta. 25 Hz smoothing condition.*

For the original data, the pattern of correlations across the width of analysis windows is the same as in the 12 Hz condition (η = 0.8 > η = 0.2 > η = 0.08 > global) with pairwise differences are highly significant (*p* < 0.001) in a sign test. For the surrogate data, however, there are no significant differences between values of η, though all of the CMA conditions show significantly higher magnitude correlations than the overall.

#### Lag Analysis

**FIGURE 7:**
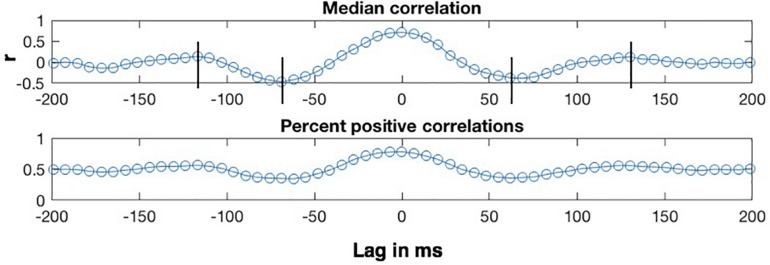
Sample results of CMA lag analysis for one speaker for correlation between MBEAM and MFCC modulation functions filtered at 12 Hz with η = 0.8. **(Top)** Shows the median value of the correlation function for each signal lag in ms. Vertical lines indicate correlation minima and secondary maxima. **(Bottom)** Shows the percentage of correlation values that are positive at each lag.

The lag analyses were conducted on the η = 0.8 condition, which exhibits the highest correlations. The top panel of Figure 7 shows how the median of the CMA correlation function varies as a function of the lag between the MBEAM and MFCC functions for one speaker, for lags between +200 and −200 ms. Positive lags represent delay of the MBEAM signal with respect to the MFCC, and negative lags represent relative delay of the MFCC function. The lower panel shows the percentage of values in the correlation function at a given lag that are positive. The two functions of lag track each other quite closely, and the analysis will focus on the median correlation lag function. Even though the figure represents correlation of two different signals, it has the form of an autocorrelation function. Very high values are found at lag = 0, in this case 0.71 (Of course, if this were an actual autocorrelation function, the value would be equal to 1 at lag = 0). As the signals are shifted in time, the correlation decreases to minimum values at lags (± 65 ms), and then increases again to maxima between 100 and 150 ms of shift (in either direction). The surrogate data did not in general exhibit this kind of structure and was not considered further in the lag analysis.

Crucially, the fact that there are secondary maxima means that there is a repetitive period in the signals that is shared between them, just as the secondary maxima in autocorrelation can be used to determine the major periodicity of a single signal. Twenty-one of the twenty-three speakers exhibit these second maxima. The lag values at which the secondary maxima occur for a given speaker were determined as follows. First, the lags corresponding to correlation minima were determined by analyzing the median correlation lag function and finding the negative extrema closest to lag = 0. Then, the secondary maxima were found by finding a maximum between the time of the minima and + or −170 ms. Since the function was noisy around the secondary maxima for several speakers, there were sometimes multiple nearby maxima in which case the most extreme one was chosen. The lag values at which the secondary maxima occurred for a given speaker were referenced to the lag value exhibiting the (primary) maximum. This is lag = 0 for the speaker shown in Figure 7, but this varied across speakers with a median value of 6.866 ms, or a delay of MBEAM by one frame. The measured lag was subtracted from the lag at which the primary maximum occurs. The positive lag and the absolute value of the negative lag were averaged to derive a single secondary maximum lag value for each speaker.

**FIGURE 8:**
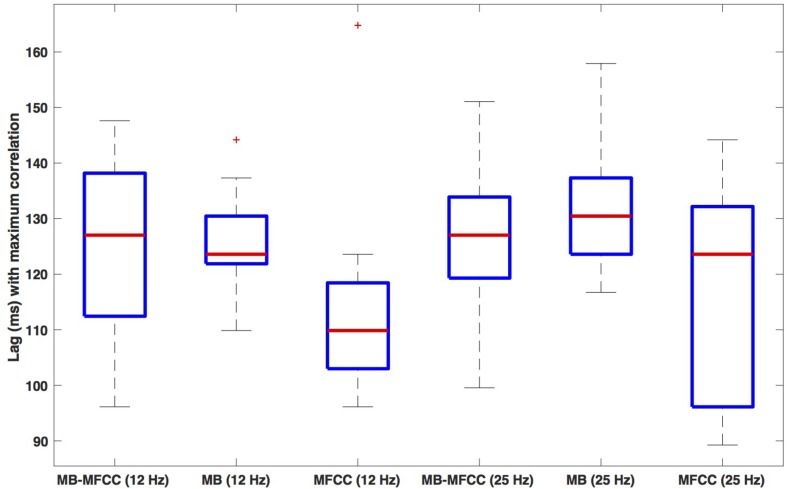
Box plots of the lag value (in ms) at which secondary maxima of correlation function are found. Lags are the average of the absolute values of the negative and positive lags. The three leftmost plots show (respectively): the lags for correlation of MBEAM with MFCC, the autocorrelation of MBEAM, and the autocorrelation of MFCC, all for the 12 Hz filtering condition. The three rightmost plots show the same three signal correlations for the 25 Hz filtering condition.

Box plots of the lag of the secondary maxima are shown in Figure 8. The leftmost plot shows the lags for the MBEAM-MFCC correlation for the 12 Hz filtering condition. The median value is 127 ms, which is very close to the median duration of the MBEAM inter-pulse intervals (132 ms). The next box plot shows the secondary maxima lags of the MBEAM function with itself (autocorrelation), with a median value of 124 ms, very close to the value for the MBEAM-MFCC correlation (though the values MBEAM-MFCC are more variable across speakers). This indicates that there is a repetitive structure to MFCC modulation function that aligns with the repetitive structure of the MBEAM function, even though the median inter-pulse interval for the MFCC function is actually shorter (116 ms), as is the median of the secondary maxima lags of the autocorrelation of the MFCC (110 ms). These differences are small in magnitude, to be sure, but the next three box plots from the 25 Hz filtering condition show the same pattern with a much larger magnitude. The MBEAM-MFCC correlation shows a median secondary maximum lag at 127 ms, similar to the median duration of the MBEAM inter-pulse intervals in this condition, 131 ms. However, the median duration of the MFCC inter-pulse intervals in this condition is 63 ms. This suggests that the MBEAM pulses are aligning with approximately every other MFCC pulse in this condition.

## Discussion

The results of the analyses provide support for the primary hypothesis that there are robust correlations between the acoustic and articulatory modulation functions, as instantiated here in the MFCC and MBEAM functions [prediction (3) in the Introduction]. On the one hand, it is not surprising that they should be correlated given their causal relationship, but there are several reasons why these particular functions might not have revealed that. Primarily, there are several articulatory dimensions of change that are not represented in the microbeam data, including information about the velum, glottis, and pharynx. The lack of such information may be part of the reason that the pulses in the MFCC function were observed to have a considerably higher frequency than those of the MBEAM function (in addition to the intrinsic smoothness of articulatory movement), particularly when the MFCC function is not low-pass filtered at the 12 Hz frequency that appears to be the highest frequency in the MBEAM function. So, the fact that significant correlations are observed even in the 25 Hz filtering condition, where the pulse frequencies are quite different, is testament to the robustness of the co-modulation effect. Another indicator of its robustness is that fact that the correlation values are so consistent across speakers. Almost all speakers show predominantly positive correlations with maximal correlations close to zero lag, and the differences across various conditions tested were generally highly significant in simple sign tests, meaning all or almost all of the speakers showed differences in the same direction. The surrogate data show highly variable correlations across speakers in both sign and lag. This is consistent with the idea that correlations in the original data are intrinsic to the physics in combination with the phonological structure and are not parameters that set differently by individual speakers. Also, the fact that robust correlations can be found in narrow time windows indicates that the correlations are not dependent on including long enough stretches of speech such as to include systematic variation in articulator velocity due to prosodic boundaries.

The lag analysis revealed that pulse sequences of the articulatory and acoustic modulation functions share a repetitive structure (prediction 4), even when the MFCC function was twice the frequency of the MBEAM function. Returning to the issue raised in the introduction of how sensory and motor representations could be aligned within the nervous system, this result supports the possibility of modulation functions contributing to the solution. Rhythmic properties of articulatory modulation could entrain oscillations in speech-motor areas, and acoustic modulation could entrain oscillations in auditory areas. The correlations of the modulation functions demonstrated in the results could contribute therefore to auditory-motor synchronization. The correlations are high and are also sensitive to lag, so oscillations in motor and auditory areas, entrained respectively to articulatory and acoustic modulation functions would tend to be in-phase and effectively synchronized. One way to quantify the sensitivity to lag is to find the threshold lag at which the percentage of positive correlations in the correlation function drops to under 50%. For the η = 0.8 (12 Hz filtering condition), the median threshold across speakers is ∼40 ms. This means that the auditory and speech motor cortical oscillations based on these respective acoustic and articulatory modulation functions would intrinsically be within 40 ms of being in phase during speech production. Coupling between activity in these brain areas, as demonstrated during listening by Assaneo and Poeppel (2018) could further strengthen the synchronization.

The approach used here to reveal the shared repetitive stricture was somewhat indirect and limited, in that ultimately it was based on a linear correlation method. A better analysis that would avoid this limitation would to use a larger corpus of material and possibly a technique like joint recurrence analysis (Marwan et al., 2007; Lancia et al., 2019). Another alternative method that avoids the linear correlation would be to measure mutual information (Cover and Thomas, 2006) between the modulation functions. Mutual information measures how much knowledge of one signal reduces uncertainty about the other, and does not depend on linear correlations. Other possible methods of looking at temporal co-modulation, based on work with neural oscillations, would deploy frequency coupling (e.g., between faster and slower frequencies) or cross-wavelet power (Grinsted et al., 2004) between modulations of acoustics and articulation in different frequency bands.

The two other predictions about the structure of the modulation functions and their relation to syllable structure were supported by the analyses presented. The modulation functions have a repetitive pulse-like structure (prediction 1). The pulse structure appears to be related to syllable structure (prediction 2). On average one pulse was found for simple CV syllables, approximately 1.5 for syllables with a coda consonant, and 2.5 for syllables with multiple coda consonants. Of course, this needs to be tested on a larger and more varied corpus, particularly including syllables with multiple onset consonants. To the extent that such future analyses support the preliminary results obtained here, it may be possible to develop a new fully spatiotemporal model of syllable structure based on kinetic energy (of the articulators or the spectrum), departing from previous models that are either purely temporal (Goldstein et al., 2006; Nam et al., 2009) or purely spatial (i.e., sonority-based^3^, for example, Goldsmith and Larson, 1990).

While such a model of syllable structure would have several attractive features, its development would require systematic investigation of a wide variety of syllable structures and their resulting kinetic energy functions. A few speculations are nonetheless merited here. While kinetic energy is not an index of sonority *per se*, it could be an index of *sonority change*, such that a sharp sonority cline (the cross-linguistically preferred syllable onset or coda pattern) is indexed by a high magnitude of the kinetic energy pulse. Also, sequences of consonants in onset or coda that obey the sonority sequencing principle might result in single modulation pulses, while those that run counter to it could exhibit multiple pulses. Which is to say, a preference for single, high-magnitude pulses capable of entraining theta oscillations could underlie the preferred syllable structures in languages. Similar computations over sonority are the basis of Goldsmith and Larson’s (1990) dynamical model of syllabification, but the values of sonority in that model are stipulated rather than representing measurable properties of speech, and temporal properties are not considered.

This modulation pulse model might also be able to provide insight into syllabification in languages in which syllables without vowels are common, such as Tashliyt Berber (Dell and Elmedlaoui, 1985) or Moroccan Arabic (Dell and Elmedlaoui, 2002). Data on articulatory organization of such vowel-less syllables has shown that the sequence of consonants constituting the onset and nucleus are organized such that the constriction gesture for the first consonant is fully released before the second is formed (Goldstein et al., 2006, for Tashhiyt; Gafos et al., 2019, for Moroccan Arabic). The sequential production of the two gestures could produce a modulation pulse that might be lacking if the two gestures were coordinated in a temporally overlapping pattern. Finally, the modulation pulse model might be able to distinguish glides (like /j/) from their corresponding vowels (like /i/), even though they are phonetically very similar in terms of static articulatory and acoustic properties. In standard phonological theory, the difference emerges as a function of being ‘parsed’ into the onset versus nucleus. In a modulation pulse model, this difference could emerge due to different patterns of overlap of an initial consonant gesture with a following glide (/Cj/) versus an initial consonant gesture with a following vowel (/Ci/). The overlap pattern in  /Ci/  would presumably produce a modulation pulse (as it does in the data analyzed here), but the overlap pattern in /Cj/could fail to add a distinct modulation pulse.

## Conclusion

While there is abundant empirical evidence for real-time sensorimotor interaction in speech production and perception, not the least of which is its requisite status in vocal learning and development, the patterns of neural activation associated with articulation and with acoustics of the same utterance are in fact distinct. This raises the question of the nature and basis of the neural binding that affords their integration. This paper presents a novel approach to this question by explicitly considering the temporal aspects of continuous acoustic and articulatory signals, which must of physical necessity be lawfully related, as the articulatory movements actually cause the acoustic signals. We hypothesize that the systematic relation between the temporal modulation of articulation and the corresponding temporal modulation of the acoustic signal offers the basis—or at least one critical basis—for the binding of production and perception, offering here an initial systematic and quantitative, albeit exploratory, investigation of the structure of the co-modulation patterns in articulation and acoustics. This preliminary data analysis identifies a pulse-like modulation structure related to syllable structure that is aligned systematically between oral articulatory movements and acoustic mfccs. Temporal co-modulation of articulation and acoustics can provide a springboard for illuminating the binding of language production and perception and its cognitive significance in phonological structuring.

## Data Availability Statement

The data analyzed in this study were obtained from the NIH-funded University of Wisconsin X-ray Microbeam project directed by John Westbury. Requests to access these datasets should be directed to John Westbury (john.westbury@wisc.edu). The results of the analyses performed here will be made available by the author, without undue reservation, to any qualified researcher upon request.

## Author Contrbutions

The author confirms being the sole contributor of this work and has approved it for publication.

## Conflict of Interest

The author declares that the research was conducted in the absence of any commercial or financial relationships that could be construed as a potential conflict of interest.

## Footnotes

1 https://www.mathworks.com/matlabcentral/fileexchange/32849-htk-mfcc-matlabh

2 We might expect that zero lag would result in the highest correlations, but because of the temporal advance of MFCC function due to the size of its analysis window as discussed earlier, this is not always the case. For example, the median of the lags that show the highest correlation for the η = 0.8 condition is 6.866 ms: the MBEAM function is delayed by one frame.

3 “Sonority” has never been given a precise physical definition, but has been approximately described as the relative “acoustic energy” of a segment (Ladefoged, 1993). In general, relative sonority of segments within a syllable increases from onset to nucleus and decreases again from nucleus to coda.
